# Bilateral living-related lobar lung transplantation avoiding bronchial stenosis associated with scoliosis by modified anastomosis: a case report

**DOI:** 10.1186/s44215-023-00089-4

**Published:** 2023-08-25

**Authors:** Junichi Takada, Masaaki Sato, Chihiro Konoeda, Hiroyuki Kaneko, Shogo Shimada, Yasutaka Hirata, Erika Yuasa, Takayuki Oyanagi, Hiroyuki Fukushima, Jun Nakajima

**Affiliations:** 1 Department of Thoracic Surgery, The University of Tokyo Graduate School of Medicine, 7-3-1 Hongo, Bunkyo-Ku, Tokyo 113-8655 Japan; 2 Department of Pediatrics, Keio University School of Medicine, 35 Shinano-Machi, Shinjuku-Ku, Tokyo 160-8582 Japan

**Keywords:** Anastomosis, Bronchial stenosis, Lung transplantation, Scoliosis

## Abstract

**Background:**

Severe thoracic deformity caused by scoliosis often hampers lung transplantation (LTx) due to its underlying restrictive ventilatory dysfunction. Postoperative stenosis of the reconstructed bronchus due to spinal compression is also a complication after LTx in patients with scoliosis. Previous reports on LTx with scoliosis and its outcomes have not discussed the technical aspect of bronchial anastomosis. This report describes a case in which bronchial stenosis was avoided by modifying the angle of the right bronchial anastomosis.

**Case presentation:**

A 26-year-old woman with atrial septal defect (ASD), pulmonary hypertension, Eisenmenger’s syndrome, and severe right scoliosis underwent bilateral living-related lobar LTx with her parents as donors followed by ASD closure. Left pneumonectomy and anastomosis preceded. On the right side, after pneumonectomy, the recipient’s right main bronchus was trimmed to rotate the bronchial anastomosis clockwise by 45°. This resulted in clockwise rotation of the graft (the lower lobe of the mother’s right lung), making axes of the graft and deformed vertebrae parallel. Postoperative bronchoscopy 2 months after surgery showed no evidence of compression or stenosis of the basal bronchial branch.

**Conclusion:**

By obliquely trimming the recipient’s right main bronchus to make the angle of the pulmonary graft and deformed thorax parallel, postoperative bronchial stenosis owing to severe scoliosis was successfully avoided.

## Background

Pulmonary hypertension is a common indication for bilateral lung transplantation (LTx). Scoliosis frequently complicates pulmonary hypertension caused by congenital heart disease, such as atrial septal defect (ASD) [1]. Severe thoracic deformities such as scoliosis are considered relative contraindications to LTx [2, 3]. This is primarily due to the fact that restrictive ventilation problems caused by thoracic deformity cannot be corrected solely by replacing the lungs.

In addition to restrictive ventilation, severe scoliosis has been linked to posttransplant airway compression due to protrusion of the vertebra into the thoracic cavity. Some reports have indicated LTx surgery in patients with scoliosis [4–6]. Numerous patients developed bronchial stenosis after surgery and required endoscopic dilatation more than once [4, 6]. We recently performed bilateral lobar LTx in a patient with Eisenmenger’s syndrome secondary to ASD. To avoid the aforementioned postoperative airway complication, we performed right bronchial anastomosis by obliquely trimming the recipient’s bronchus to rotate the graft clockwise and align the axes of the graft and the deformed vertebrae.

## Case presentation

A 26-year-old woman had been diagnosed with ASD, pulmonary hypertension, and severe right scoliosis since her neonatal period. She developed Eisenmenger’s syndrome and was suffering from right heart failure, which was managed by medical treatment, including continuous intravenous administration of epoprostenol (eventual dose, 50 ng/kg/min), oral endothelin receptor antagonists (eventual dose of macitentan, 3 mg/day), and beta-blockers; phosphodiesterase inhibitors were discontinued due to its side effects. Although she was referred to another transplantation center approximately 10 years ago, she was deemed unsuitable for LTx because her scoliosis and chest deformity were extremely severe and her quality of life was good. However, her condition rapidly deteriorated with signs of left heart failure; her echocardiogram revealed left ventricular outflow tract stenosis (LVOTS) and severe mitral valve regurgitation (MR) associated with systolic anterior movement of mitral valve (SAM), which was secondary to exacerbation of pulmonary hypertension along with hyperdynamic state probably due to epoprostenol. Her hemodynamics were stabilized by the administration of catecholamines and decreasing doses of epoprostenol (down to 40 ng/kg/min), which resulted in resolution of LVOTS and MR with apparently good left ventricular function. Isolated LTx with ASD closure instead of combined heart–lung transplantation was considered to be enough to save her life. LTx from a deceased donor was ruled out because of the long waiting period in Japan (approximately 3 years) [7]. Thus, living-related LTx and ASD closure were the only realistic option. We carefully evaluated the patient’s candidacy for living-related lobar LTx, while her parents and the donor candidates underwent medical examinations to determine their suitability as donors.

**Fig. 1:**
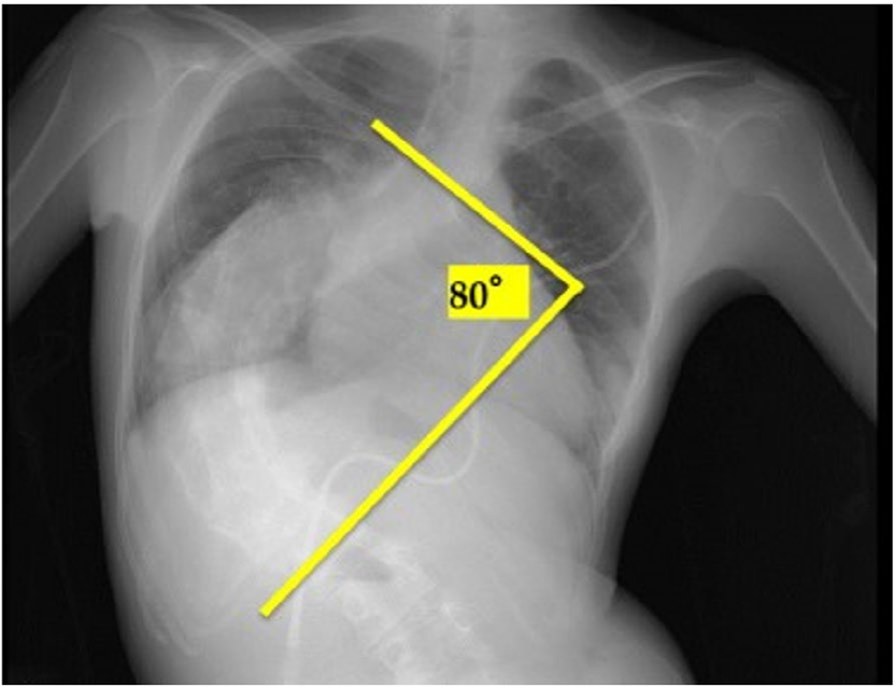
Severe scoliosis shown in preoperative chest X-ray. The Cobb’s angle (the greatest angle between the vertebrae) is 80°. This is the case of a patient with severe scoliosis who is generally recommended to receive orthopedic surgery

**Fig. 2:**
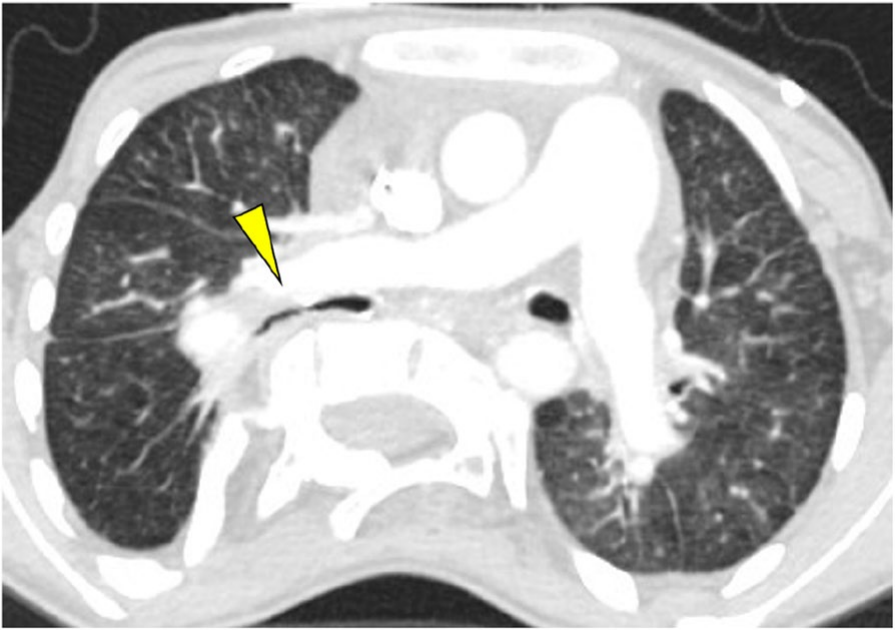
Preoperative right bronchial stenosis due to vertebral compression. Before LTx, the right main bronchus is stenosed by posterior compression of the scoliotic vertebra

Chest X-ray revealed severe scoliosis with Cobb’s angle, the largest angle measured between two different vertebrae, of 80° (Fig. 1). Computed tomography (CT) confirmed bronchial stenosis due to vertebral compression at the level of the right main bronchus (Fig. 2). Furthermore, atrial blood gas test revealed PaO_2_ of 48 mmHg and PaCO_2_ of 39.4 mmHg under conditions of 21% FiO_2_ without respiratory assistance. Despite the presence of hypoxemia mainly due to right-to-left shunt, there was no tendency toward CO_2_ retention. Echocardiography revealed a right ventricular systolic pressure of 88 mmHg with normal left ventricular function (ejection fraction, 64%).

**Fig. 3:**
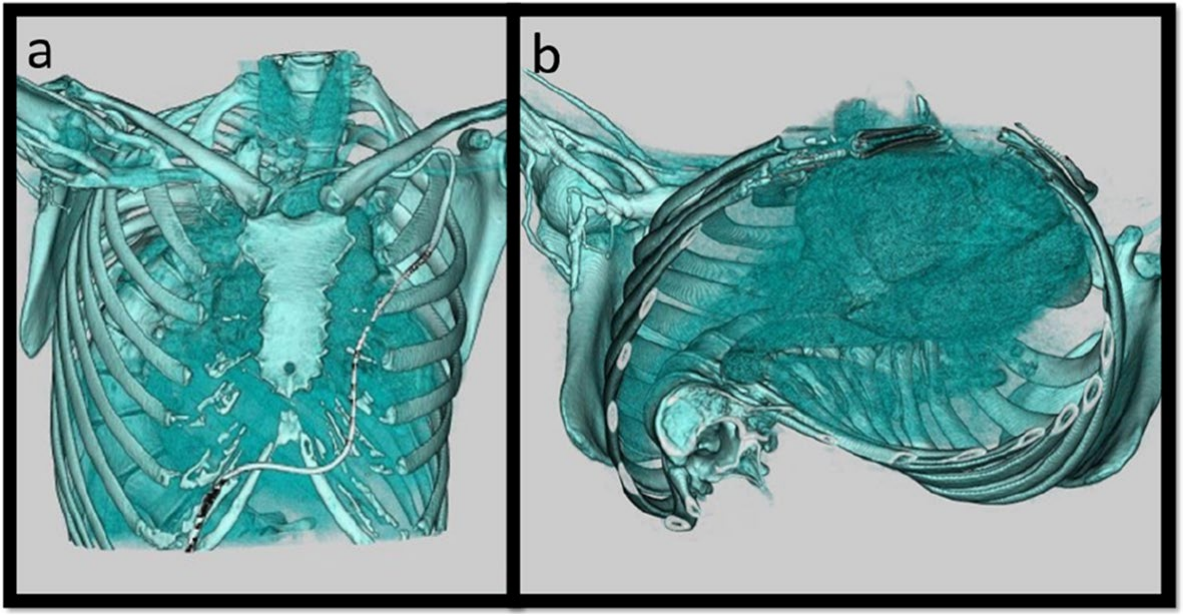
Preoperative 3D-CT revealed chest deformity. **a** Frontal view: her anterior chest anatomy was normal compared with posterior scoliosis, suggesting the use of regular clamshell approach. **b** View from the diaphragm: her vertebrae shifted to the right side due to scoliosis. The right thorax was relatively smaller than the left

Based on the above findings, we determined that her restrictive ventilation impairment due to thoracic deformity was not severe enough to rule her out of transplant candidacy, despite the fact that the risks associated with LTx were extremely high. Her chest deformity was also examined using 3D-CT, which demonstrated relatively normal anterior chest anatomy, suggesting the use of regular clamshell approach (Fig. 3).

We employed 3D-CT volumetry to perform preoperative size matching. Because the right thorax was relatively small due to scoliosis, a smaller graft of the mother’s right lower lobe (predicted volume: 837 mL) was selected for the right side, and a larger graft of the father’s left lower lobe (predicted volume: 888 mL) was selected for the left side. The total graft volume (1725 mL) was calculated as 62.3% of the recipient’s bilateral thoracic volume, which was considered appropriate for living-related lobar LTx.

**Fig. 4:**
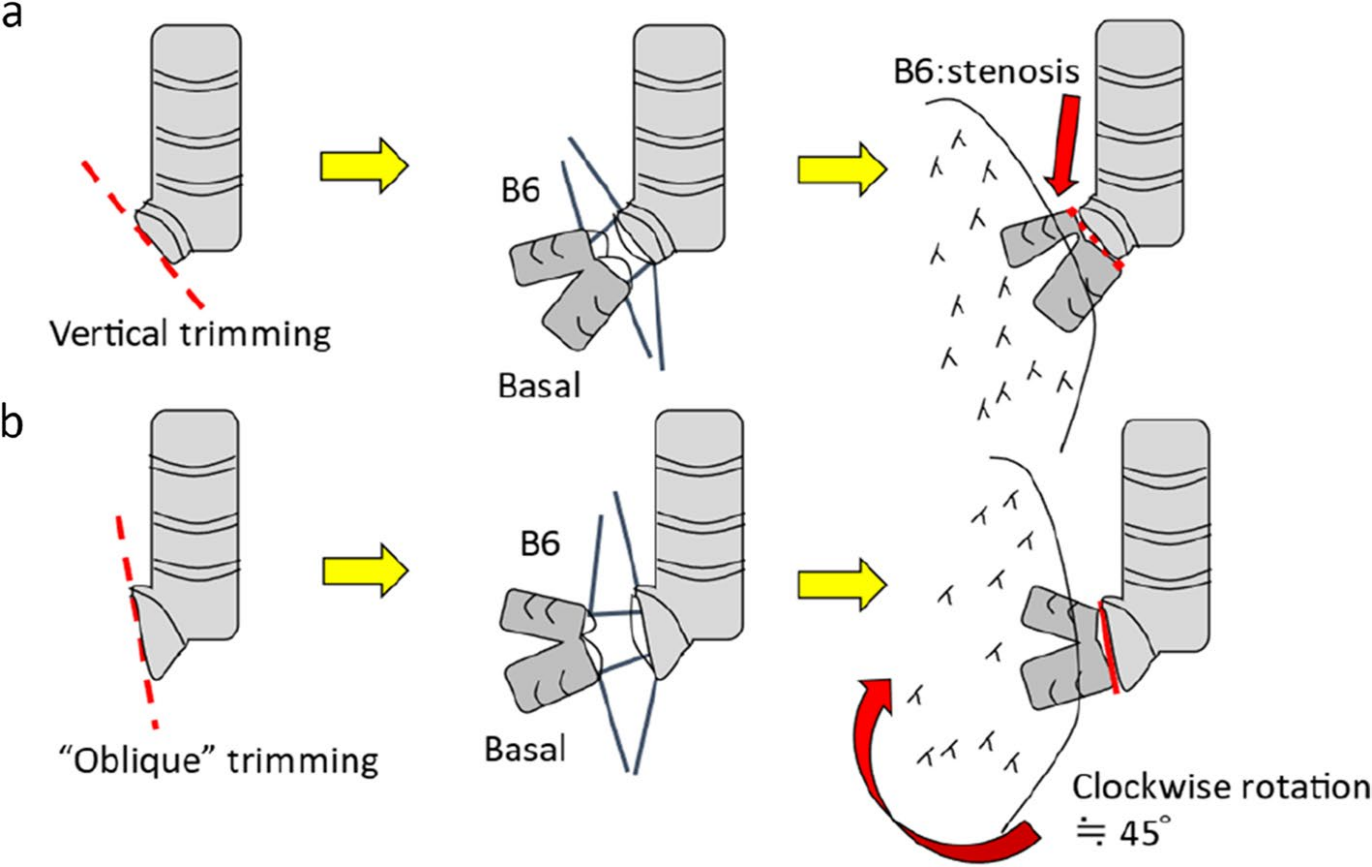
The modified angle of bronchial anastomosis. **a** The bronchi are usually trimmed vertically, and end-to-end anastomosis is performed. **b** Our “oblique trimming” with the cephalic line of the recipient bronchus deviated toward the trachea will avoid compression by the vertebra and stenosis of the B6 bronchus, resulting in fitting of the graft to the deformed thorax

**Fig. 5:**
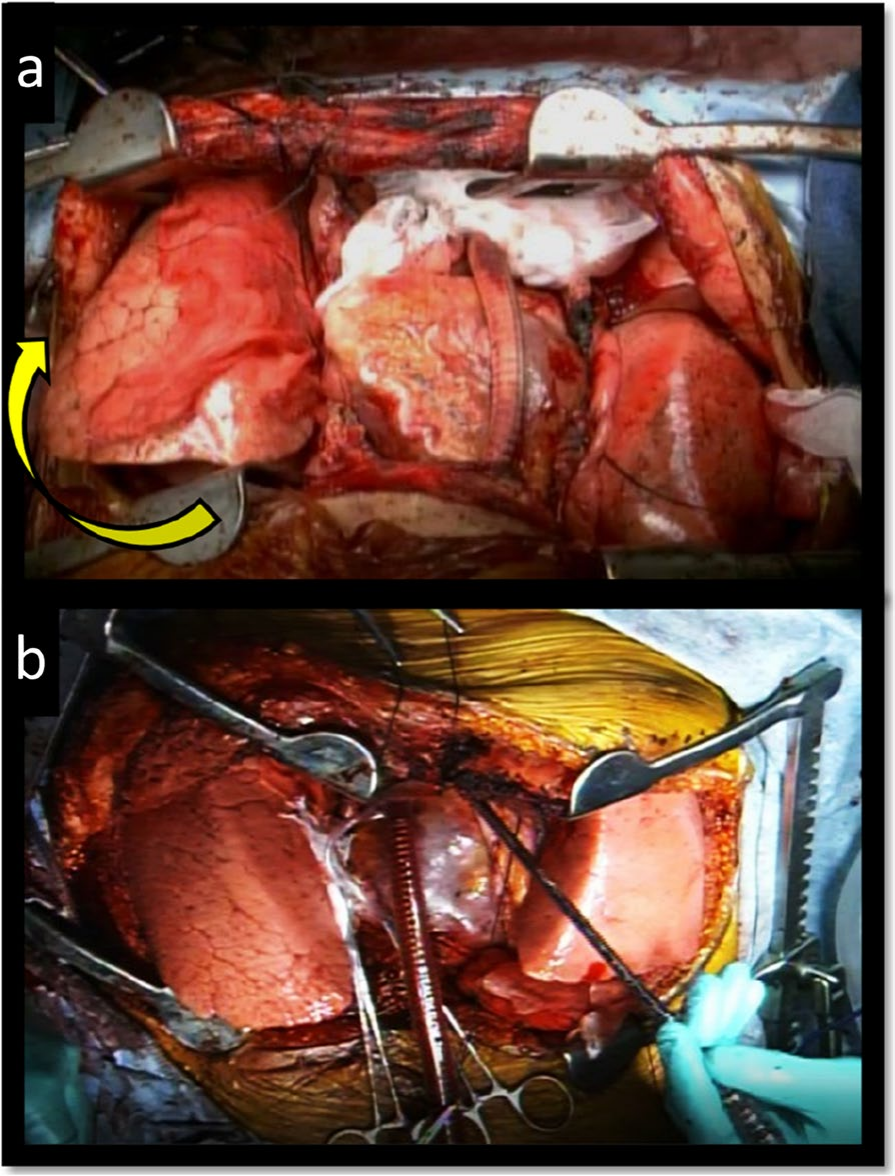
Clockwise rotation of the right graft. **a** The right graft rotates clockwise by approximately 45° with good inflation. **b** A normal LTx case as a reference for comparison

The operation was performed as follows. A clamshell thoracotomy along the fourth intercostal space was performed with the patient in the supine position under general anesthesia. The anterior thorax was not affected by scoliosis, and a normal clamshell incision was made. Under extracorporeal membrane oxygenation (ECMO), left pneumonectomy and anastomosis were performed. Subsequently, right pneumonectomy was performed. The recipient’s right main bronchus was trimmed to rotate the bronchial anastomosis clockwise by 45° (Fig. 4). This caused the graft (the lower lobe of the mother’s right lung) to rotate clockwise, making the axes of the graft and deformed vertebrae almost parallel in the thorax (Fig. 5). Next, the donor’s right inferior pulmonary vein was anastomosed to the recipient’s right superior pulmonary vein, followed by anastomosis of the pulmonary artery in a regular manner. Finally, after switching to a cardiopulmonary bypass, the ASD was closed by direct suture. The total operative time was 12 h and 38 min, with a blood loss of 1625 mL.

**Fig. 6:**
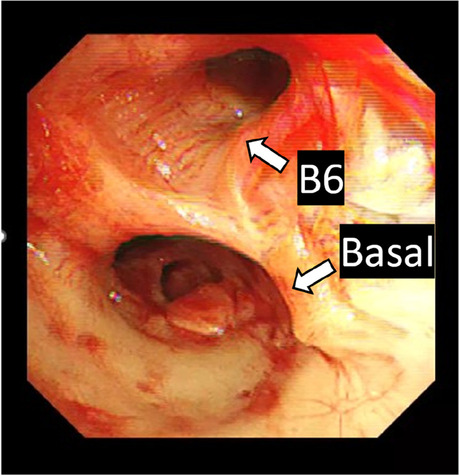
Bronchoscopic image of distal to right anastomosis 2 months after LTx. The right basal bronchial branch is open, and its lumen coloration is good. No findings were suggestive of stenosis

After surgery, a cerebral infarction due to thrombotic occlusion of the periphery of the right middle cerebral artery was confirmed using CT, which was indicated because the bispectral index monitored by anesthesiologists suddenly dropped toward the end of surgery. Although she did not suffer from obvious neurological sequelae, such as paralysis, she exhibited impaired swallowing function and muscle weakness. In addition, she had restrictive ventilation impairment and exhibited a tendency to CO_2_ retention, probably because of thoracic deformity along with postoperative muscle weakness. On the other hand, her hemodynamics were consistently stable without any signs of heart failure. She was weaned off ECMO in 1 day and catecholamines in 2 weeks, allowing for adequate physical and respiratory rehabilitation. After 4 months of rehabilitation, she achieved complete weaning from the ventilator. She was finally discharged home 7 months after surgery. Moreover, although acute cellular rejection temporarily reduced her pulmonary function, which required one steroid pulse therapy, it gradually recovered. Bronchoscopy 2 months after LTx showed no evidence of stenosis or obstruction of the right basal bronchial branch (Fig. 6). Overall, we believe that her LTx surgery strategy, including the technical aspect of bronchial anastomosis, was successful.

## Discussion and conclusions

Herein, we reported our experience with bilateral living-related lobar LTx in a patient with pulmonary hypertension caused by ASD and complicated by severe scoliosis. We obliquely trimmed the recipient’s bronchus and adjusted the axis of the graft before the right bronchial anastomosis to avoid compression from the thoracic vertebrae, thus preventing postoperative airway complications.

Severe scoliosis worsens respiratory function in two aspects. First, as observed in the present case (Fig. 3), severe scoliosis is associated with 3D deformity of the thoracic cavity [8]. It reduces thorax mobility and misaligns respiratory muscles during the breathing motion, resulting in restrictive ventilation impairment [9]. Therefore, severe thoracic deformity is generally regarded as a relative contraindication to LTx. In this case, another transplantation center did not consider her a suitable candidate for LTx 10 years ago because of her severe scoliosis and good quality of life at that time. When we considered LTx, she was suffering from severe pulmonary hypertension, which had not improved despite maximal medical treatment. Accordingly, we believed that living-related lobar LTx was the only realistic alternative for saving her life in Japan, where deceased donors were insufficient. Notably, because correcting the deformity of the entire thorax at the time of LTx is challenging, restrictive ventilation impairment persists after LTx. In addition to rehabilitation, as in this case, orthopedic surgery can be performed following LTx to treat scoliosis and resolve ventilation problems. Shiraishi et al. reported a case in which a patient with progressive scoliosis and restrictive ventilation impairment underwent spinal fusion surgery and achieved complete weaning from the ventilator 7 years after LTx [10].

Second, as shown in the preoperative CT image of this patient (Fig. 2), the thoracic vertebrae protruding into the thoracic cavity can directly compress the bronchi (mostly on the scoliotic side) from behind, causing airway stenosis [11]. Several reports have indicated stenosis of the reconstructed bronchi after LTx in patients with thoracic deformity due to scoliosis [4–6]. However, to the best of our knowledge, no reports have been published on intraoperative techniques that focus on the angle of anastomosis. This could be due to the fact that standard bronchial anastomosis procedure can be performed even with scoliosis. We believe that such reconstructed airway compression can be avoided by considering a particular angle of anastomosis using our “oblique trimming” technique.

The technique was based on previous reports. Regarding LTx, Weder et al. reported that an oblique cut of the donor bronchus is important to avoid complications [12]. Moreover, oblique bronchial trimming is sometimes performed during extended sleeve lobectomy for advanced central-located lung cancer, with double or more lobectomy performed simultaneously [13, 14]. Such trimming overcomes the difference in caliber between the central main bronchus and peripheral bronchi at the anastomosis [15]. Compared with the more common method of sleeve lobectomy, which involves vertical trimming of the bronchus, this technique shows generally acceptable outcomes, with no increase in anastomosis-related complications or inhospital mortality [13–15].

Another characteristic of the presented operative technique was to fully use the apical thoracic space, which is often left unfilled in living lobar transplantation due to differences in graft and thorax shapes. In this case, by rotating the anastomosis and thus the lobar graft, the apical space was better filled without causing atelectasis on the scoliotic side (Fig. 5). These technical devices, which we developed for living lobar LTx, are also applicable to deceased-donor LTx in patients with severe scoliosis. In particular, a larger donor graft is selected compared with the recipient organ, followed by lobar transplantation only on the scoliotic side. By rotating the graft as demonstrated in this case, bronchial stenosis could be avoided, and the recipient’s mismatched small thoracic cavity on the scoliotic side could be better filled with the graft. For the contralateral side, where a larger thoracic space is available due to the missing vertebral body, a relatively large whole lung graft may be fixed.

In conclusion, it was critical to perform the scoliotic side bronchial anastomosis in cases of LTx with scoliosis by obliquely trimming the recipient’s bronchus to rotate the graft and make the axes of the graft and the deformed vertebrae parallel, thereby avoiding postoperative airway complications.

## Data Availability

Not applicable.

## Abbreviations

LTx: Lung transplantation
ASD: Atrial septal defect
CT: Computed tomography
LVOTS: Left ventricular outflow tract stenosis
MR: Mitral valve regurgitation
SAM: Systolic anterior movement of mitral valve
ECMO: Extracorporeal membrane oxygenation
